# Sleep Characteristics and Risk of Stroke and Dementia: An Observational and Mendelian Randomization Study

**DOI:** 10.1212/WNL.0000000000209141

**Published:** 2024-02-13

**Authors:** Chutian Guo, Eric L. Harshfield, Hugh S. Markus

**Affiliations:** From the Stroke Research Group, Department of Clinical Neurosciences, University of Cambridge, United Kingdom.

## Abstract

**Background and Objectives:**

Sleep disturbances are implicated as risk factors of both stroke and dementia. However, whether these associations are causal and whether treatment of sleep disorders could reduce stroke and dementia risk remain uncertain. We aimed to evaluate associations and ascertain causal relationships between sleep characteristics and stroke/dementia risk and MRI markers of small vessel disease (SVD).

**Methods:**

We used data sets from a multicenter population-based study and summary statistics from genome-wide association studies (GWASs) of sleep characteristics and outcomes. We analyzed 502,383 UK Biobank participants with self-reported sleep measurements, including sleep duration, insomnia, chronotype, napping, daytime dozing, and snoring. In observational analyses, the primary outcomes were incident stroke, dementia, and their subtypes, alongside SVD markers. Hazard ratios (HRs) and odds ratios (ORs) were adjusted for age, sex, and ethnicity, and additional vascular risk factors. In Mendelian randomization (MR) analyses, ORs or risk ratios are reported for the association of each genetic score with clinical or MRI end points.

**Results:**

Among 502,383 participants (mean [SD] age, 56.5 [8.1] years; 54.4% female), there were 7,668 cases of all-cause dementia and 10,334 strokes. In longitudinal analyses, after controlling for cardiovascular risk factors, participants with insomnia, daytime napping, and dozing were associated with increased risk of any stroke (HR 1.05, 95% CI 1.01–1.11, *p* = 8.53 × 10^−3^; HR 1.09, 95% CI 1.05–1.14, *p* = 3.20 × 10^−5^; HR 1.19, 95% CI 1.08–1.32, *p* = 4.89 × 10^−4^, respectively). Almost all sleep measures were associated with dementia risk (all *p* < 0.001, except insomnia). Cross-sectional analyses identified associations between napping, snoring, and MRI markers of SVD (all *p* < 0.001). MR analyses supported a causal link between genetically predicted insomnia and increased stroke risk (OR 1.31, 95% CI 1.13–1.51, *p* = 0.00072), but not with dementia or SVD markers.

**Discussion:**

We found that multiple sleep measures predicted future risk of stroke and dementia, but these associations were attenuated after controlling for cardiovascular risk factors and were absent in MR analyses for Alzheimer disease. This suggests possible confounding or reverse causation, implying caution before proposing sleep disorder modifications for dementia treatment.

## Introduction

Sleep disorders have been suggested as a causal risk factor of both stroke and dementia.^1,2^ Studies investigating associations of sleep have investigated several sleep phenotypes including sleep duration, sleep chronotype, insomnia, napping, daytime dozing, and snoring. Many associations with these measures have been reported: Both short and long sleep duration are associated with increased risk of overall cardiovascular disease mortality,^3^ insomnia is associated with cerebral small vessel disease (SVD) risk,^4^ and both short sleep duration and insomnia are associated with increased dementia risk.^5,6^

Most previous studies investigating sleep associations with stroke and dementia have been cross-sectional, which leaves open questions regarding causality. The previously identified associations with sleep could be due to confounding with vascular risk factors such as smoking and alcohol, or could arise from reverse causation, in which subclinical cerebrovascular disease or dementia causes sleep disturbance.

Evidence supporting a causal relationship can be obtained from longitudinal studies evaluating whether risk factors at baseline predict incident stroke and dementia and by using Mendelian randomization (MR). MR is a statistical approach that uses genetic variants as instrumental variables to infer the causal effect of a modifiable exposure (risk factor) on a health outcome (e.g., stroke or dementia).^7^

To investigate the role of sleep characteristics on stroke and dementia risk, we performed longitudinal analyses in over 500,000 individuals from UK Biobank and examined whether 6 different sleep measures predicted incident stroke (all stroke [AS], ischemic stroke, intracerebral hemorrhage [ICH]) and dementia (all-cause dementia, Alzheimer disease [AD], vascular dementia, frontotemporal dementia [FTD]). We then performed MR to analyze the causal nature of the associations.

A recent hypothesis is that disruption of the glymphatic system may play a key role in SVD, which is a major cause of lacunar stroke, ICH, and vascular dementia. This suggests that sleep disorders might specifically increase SVD risk. To investigate, we also evaluated in 40,000 UK Biobank participants with brain imaging whether sleep measures were associated with MRI features of SVD, including white matter hyperintensities (WMHs) and markers of white matter ultrastructural damage on diffusion tensor imaging (DTI). We further examined associations between sleep and MRI markers of SVD using MR.

## Methods

### Study Population

The UK Biobank is a prospective cohort study of 502,383 participants (aged 40–69 years) recruited from 22 centers across the United Kingdom from March 2006 to October 2010.^8^ Participants completed self-reported questionnaires, verbal interviews, physical measurements, and blood sample collection. All UK Biobank protocols were approved by external ethics committees (reference 11/NW/0382), and all participants provided informed consent.^9^

### Sleep Measures

The UK Biobank recorded information using the touchscreen questionnaire on several sleep measures including sleep duration, chronotype (morning/evening person), daytime napping (short periods of sleep taken throughout the day), sleeplessness/insomnia (trouble falling asleep at night or wake up in the middle of night), daytime dozing (inability to stay awake and alert during waking hours), and snoring.

### Incident Stroke and Dementia

Clinical end points were recorded for AS, ischemic stroke, ICH, all-cause dementia, AD, vascular dementia, and FTD. These were defined based on the earliest recorded case that occurred after baseline assessment and before the end of the follow-up period. Data were obtained through self-report at a nurse interview and linkage to hospital admissions from electronic health records and death certificate records. The lists of clinical codes used to define the clinical end points were developed and validated by the UK Biobank Outcome Adjudication Group in conjunction with clinical experts.

### MRI Markers of SVD

In 2014, the UK Biobank commenced an imaging study to conduct MRI scans in a subset of ∼100,000 participants.^10^ Information for over 40,000 participants had been released at the time of this analysis. Patients with prevalent stroke were excluded. We examined WMH volume and several DTI metrics, including mean diffusivity (MD) and fractional anisotropy (FA).^11^ We log-transformed the total volume of WMHs from T1 and T2 fluid-attenuated inversion recovery images. For the DTI metrics, we performed principal component analyses on 48 markers of FA and MD derived by the UK Biobank from the FA skeleton of the diffusion MRI data, and we used the first principal component from each analysis as a summary measure.^12^ In addition, from DTI scans, we calculated peak width of skeletonized mean diffusivity (PSMD), an automated measure based on skeletonization analysis, using a published pipeline.^13^

### Genetic Instruments

Sleep measures were used as instrumental variables. We obtained genome-wide association study (GWAS) summary statistics from published analyses of UK Biobank participants for sleep duration (N = 446,118; 78 loci),^14^ chronotype (N = 697,828; 351 loci),^15^ daytime napping (N = 452,633; 123 loci),^15^ daytime dozing (N = 452,071; 42 loci),^16^ and snoring (N = 314,449; 41 loci).^17^ For insomnia, the most recent and largest GWAS summary statistics were used, with 554 genetic loci identified in 2,365,010 individuals.^18^

For outcome variables, summary statistics for stroke and ischemic stroke subtypes were obtained from participants of European ancestry from the GIGASTROKE Consortium,^19^ which consisted of 73,652 patients with stroke and 1,234,808 controls. We conducted analyses for any stroke (n = 73,652 cases), ischemic stroke (n = 62,100 cases), cardioembolic stroke (n = 10,804 cases), large-artery stroke (n = 6,399), and small vessel stroke (SVS; n = 6,811). We also used summary statistics from a cohort of neuroimaging-confirmed lacunar (small vessel) stroke, which provided more detailed phenotyping of SVS (NC_SVS, N = 6,030).^20^ Summary statistics for AD were obtained from the International Genomics of Alzheimer's Project (IGAP) (N = 21,982).^21^ For SVD imaging traits, summary statistics for WMH (N = 42,310), FA (N = 17,663), and MD (N = 17,467) were obtained from a GWAS of participants from the UK Biobank and the CHARGE Consortium.^22^ We obtained summary statistics for PSMD from a currently unpublished GWAS (N = 40,464). A summary for originating GWASs is provided in eTable 1 (links.lww.com/WNL/D422).

### Statistical Analyses

#### Cross-Sectional and Longitudinal Analyses

Ethnicity, smoking, and alcohol were coded as binary outcomes. For ethnicity, European was encoded to “0” and all other ethnicities were encoded to “1.” For smoking and alcohol, “never” was encoded to “0” while “previous” and “current” were encoded to “1.” Categorical sleep variables (insomnia, chronotype, napping, dozing) were also reconstructed as binary outcomes. For insomnia, “never/rarely” was encoded to “0” while “sometimes” and “usually” were encoded to “1.” For daytime napping and dozing, “never/rarely” was encoded to “0” while “sometimes” and “often” were encoded to “1.” For chronotype, “definitely a ‘morning’ person” and “more a ‘morning’ than ‘evening’ person” were encoded to 0, whereas “definitely an ‘evening’ person” and “more an ‘evening’ than ‘morning’ person” were encoded to 1. The associations between sleep measures and SVD imaging markers (WMH, FA, MD, PSMD) were examined in a cross-sectional analysis using linear regression models. WMH was log-transformed and the continuous outcomes were rescaled to have a mean of 0 and SD of 1. In primary analyses, we only adjusted for age, sex, and ethnicity. In secondary analyses, we adjusted for a wider range of vascular risk factors and other potential confounders (age, sex, ethnicity, body mass index, blood pressure treatment, systolic blood pressure, diastolic blood pressure, type 2 diabetes, smoking, alcohol, serum cholesterol, and Townsend deprivation index). Sensitivity analyses were performed with further adjustment for major depression and atypical antipsychotic medication usage.

Longitudinal analyses were performed to investigate whether sleep measures predicted incident stroke and dementia. In the analyses, all prevalent cases (outcomes that occurred before the baseline assessments) were excluded. Cox proportional hazards regression models were used to examine the association between sleep variables and risk of incident stroke and dementia. Primary analyses were conducted with adjustment for age, sex, and ethnicity. Secondary analyses were conducted with a wide range of vascular risk factors and potential confounders included as described for the cross-sectional analyses. Additionally, sensitivity analyses were performed with further adjustment for major depression and atypical antipsychotic medication usage and excluding all outcomes of interest that occurred within 1 year of the baseline assessment. The proportional hazards assumption was evaluated using Schoenfeld residuals.

#### MR Analyses

A 2-sample MR analysis was performed to examine whether there was evidence to support a causal relationship of sleep with stroke, dementia, and SVD imaging markers.

Primary analyses were conducted using 2-sample inverse-variance weighted univariable MR (IVW-MR). Independent genetic variants (*r*^2^ < 0.01 in European ancestry individuals in the 1000 Genomes Project, Phase 3 release [1KG]) that were associated with sleep measures at genome-wide significance (*p* < 5 × 10^−8^) were selected in European ancestry individuals. These variants were cross-referenced against the PhenoScanner database of published genetic associations to ensure that they, or their proxies (*r*^2^ ≥ 0.8 in the 1KG project), were not associated with potential confounding factors at genome-wide significance.^23^ Details of the excluded genetic variants are listed in eTable 2 (links.lww.com/WNL/D422). For all analyses, palindromic variants with ambiguous allele frequencies were discarded as were genetic variants with potential strand issues that could not be resolved. Furthermore, all variants associated with sleep measures were harmonized with the outcome data to ensure that the effect estimates of each variant on sleep and the outcome corresponded to the same-effect allele.

A range of sensitivity analyses were performed relaxing some of the stricter assumptions underlying the IVW-MR method, including the weighted median estimator, simple and weighted mode-based estimators, MR-Egger regression, and MR pleiotropy residual sum and outlier (MR-PRESSO) methods.^24^ These methods are recommended in practice for sensitivity analyses because they require different assumptions to be satisfied, and therefore, if estimates from such methods are similar, any inferred causal claims are more credible.^25^ MR pleiotropy tests were performed which examine the intercept term in MR-Egger regression to evaluate whether the result is influenced by directional horizontal pleiotropy. For significant results, reverse MR was performed to examine whether it is the case that the outcome causes the exposure. MR-PRESSO distortion and outlier tests were performed.

All observational analyses were conducted using Python 3.92 software; MR analyses were conducted in R v4.2.0 using the TwoSampleMR and MR-PRESSO packages. A false discovery rate (FDR) correction was applied with a significance threshold of 0.05.

### Standard Protocol Approvals, Registrations, and Patient Consents

All UK Biobank participants provided informed consent as part of the UK Biobank recruitment process to the use of their anonymized data and samples for any health-related research, to be recontacted for further substudies, and for UK Biobank to access their electronic health records. The UK Biobank has approval from the North West Multi-centre Research Ethics Committee as a Research Tissue Bank approval. This research was conducted using UK Biobank under application number 36509.

### Data Availability

The data supporting the findings of this study are available within the article and its Supplemental Materials. The original data from UK Biobank can be accessed by approved researchers through application to UK Biobank (ukbiobank.ac.uk/enable-your-research). The summary statistics obtained from the genome-wide association are publicly available. The summary statistics for sleep characteristics can be obtained from the Sleep Disorder Knowledge Portal (sleep.hugeamp.org/) and the Complex Traits Genetics Lab (ctg.cncr.nl/software/summary_statistics/). The summary statistics for stroke from the GIGASTROKE Consortium can be obtained from the GWAS Catalog (ebi.ac.uk/gwas/; study accession numbers GCST90104534–GCST90104563). The summary statistics for AD can be obtained from the IGAP (niagads.org/datasets/ng00075).

## Results

### Participant Characteristics

A total of 502,383 participants from the UK Biobank were analyzed, with mean age 56.5 (SD 8.1) years; 54.4% were female and 94.6% White. Details for other risk factors, sleep measures, and SVD imaging measures are summarized in Table 1.

**Table 1:** Characteristics of Participants

Characteristics	Mean (SD) or n (%)	N
Risk factors		
Female sex	273,169 (54.4)	502,011
Age at recruitment, y	56.53 (8.10)	502,383
Ethnicity (white)	472,642 (94.6)	499,667
Townsend deprivation index	−1.29 (3.09)	501,760
Body mass index, kg/m^2^	27.43 (4.80)	499,391
Systolic blood pressure, mm Hg	137.92 (18.69)	501,095
Diastolic blood pressure, mm Hg	82.24 (10.17)	501,097
Current smoker	52,969 (10.6)	499,569
Alcohol user	459,059 (91.7)	500,746
Type 2 diabetes mellitus	47,822 (9.5)	502,326
Taking blood pressure medication	103,035 (20.8)	494,987
Serum cholesterol	5.69 (1.15)	470,734
Sleep measures		
Sleep duration, h/d	7.10 (1.30)	501,514
Insomnia	141,363 (28.2)	500,898
Morningness chronotype	247,099 (55.5)	444,946
Daytime napping	219,500 (43.8)	500,508
Daytime dozing	14,092 (2.8)	498,667
Snoring	173,329 (37.2)	465,360
SVD MRI markers		
WMH volume, mm³	8.02 (1.01)	41,623
Fractional anisotropy	0.00017 (4.43)	40,746
Mean diffusivity	0.00011 (4.52)	40,746
Peak width of skeletonized mean diffusivity, mm^2^/s	0.00023 (4.1E-05)	40,464

Abbreviations: SVD = small vessel disease; WMH = white matter hyperintensity.

Mean and SD are presented for numerical variables while frequency and percentage are shown for categorical variables.

### Association of Sleep Measures With Stroke and Dementia: Longitudinal Analysis

The median number of years of follow-up was 13.07 (interquartile range [IQR] 1.40) for stroke and 13.08 (IQR 1.39) for dementia. During the follow-up period, there were 10,434 (2.07%) incident strokes, 8,785 (1.7%) ischemic strokes, 1,859 (0.37%) cases of ICH, 7,668 (1.5%) cases of all-cause dementia, 3,273 (0.6%) cases of AD, 1,736 (0.34%) cases of vascular dementia, and 264 (0.05%) cases of FTD (Table 2).

**Table 2:** Clinical End Point (Stroke and Dementia) and Cerebral SVD Data for Participants

Clinical end points	n (%)	N
Incident all stroke case	10,434 (2.07)	502,383
Incident ischemic stroke case	8,785 (1.7)	502,383
Incident intracerebral hemorrhage case	1,859 (0.37)	502,383
Incident all-cause dementia case	7,668 (1.5)	502,383
Incident Alzheimer disease case	3,272 (0.6)	502,383
Incident vascular dementia case	1,736 (0.34)	502,383
Incident frontotemporal dementia case	264 (0.05)	502,382

Abbreviation: SVD = small vessel disease.

All sleep measures, except insomnia, were associated with all-cause dementia, and all sleep measures, except snoring, were associated with vascular dementia. After adjusting for vascular risk factors, all associations with all-cause dementia remained statistically significant (all *p* < 0.01), apart from insomnia, which was no longer significant. The association of daytime dozing with vascular dementia attenuated but remained statistically significant after adjusting for vascular risk factors (hazard ratio [HR] 1.36 [1.10–1.69], *p* = 0.005). Finally, we found that insomnia (HR 0.83 [0.77–0.90], *p* = 9.50 × 10^−6^) and snoring (HR 1.11 [1.02–1.21], *p* = 0.008) were significantly associated with AD both before and after adjusting for cardiovascular risk factors.

In longitudinal analyses accounting for age, sex, and ethnicity, there were strong associations between insomnia, chronotype, daytime napping, and dozing with AS and all ischemic stroke (AIS). However, after adjusting for vascular risk factors, only insomnia, daytime napping, and dozing showed weak associations with AS (HR 1.05 [1.01–1.11], *p* = 8.53 × 10^−3^ for insomnia; HR 1.09 [1.05–1.14], *p* = 3.20 × 10^−5^ for napping; HR 1.19 [1.08–1.32], *p* = 4.89 × 10^−4^ for dozing) and AIS (HR 1.08 [1.03–1.13], *p* = 0.002 for insomnia; HR 1.11 [1.06–1.16], *p* = 4.43 × 10^−6^ for napping; HR 1.24 [1.11–1.37], *p* = 6.64 × 10^−5^ for dozing). The sensitivity analyses with further adjustment for major depression and atypical antipsychotics medication usage and excluding all outcomes of interest that occurred within 1 year of the baseline assessment did not affect any significance level. To evaluate validity of models, scaled Schoenfeld residuals global tests were performed.

**Figure 1:**
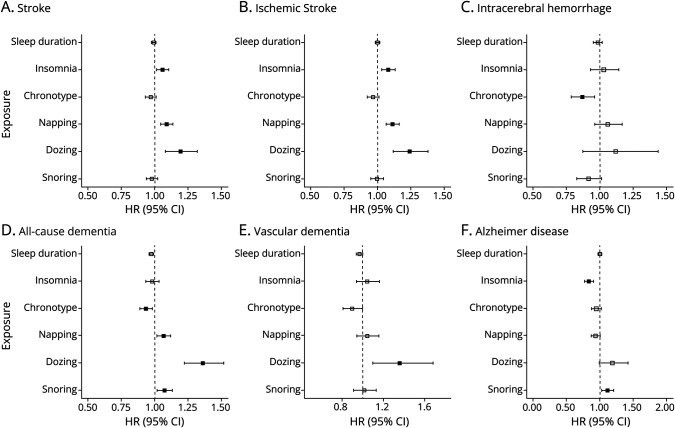
Longitudinal Analyses for the Association of Sleep Measures With Stroke and Dementia Associations are shown for sleep duration, insomnia, chronotype, daytime napping, dozing, and snoring with stroke (A–C, stroke, ischemic stroke, intracerebral hemorrhage) and dementia (D–F, all-cause dementia, vascular dementia, Alzheimer disease). The x-axis represents the hazard ratio, and solid squares indicate significant associations after FDR correction. The results are controlled for covariates including both demographic variables and cardiovascular risk factors. FDR = false discovery rate; HR = hazard ratio.

The results for longitudinal studies after adjusting for confounders and vascular risk factors are presented in Figure 1. Detailed association results are presented in eTable 3 (links.lww.com/WNL/D422) for primary analyses, eTable 4 for secondary analyses, eTables 5 and 6 for sensitivity analyses, and eTable 7 for scaled Schoenfeld residual tests.

### Association of Sleep Measures With MRI Markers of SVD: Cross-Sectional Analysis

After adjustment for sex, age, and ethnicity, daytime napping was associated with higher WMH (odds ratio [OR] 1.99, *p* = 5.56 × 10^−13^ [1.95–2.03]), FA (OR 1.24 [1.14–1.35], *p* = 7.21 × 10^−7^), MD (OR 1.17 [1.07.1.27], *p* = 2.82 × 10^−4^), and PSMD (OR 1.05 [1.03–1.07], *p* = 2.26 × 10^−8^). Furthermore, insomnia (OR 1.16 [1.06–1.27], *p* = 0.002) and snoring (OR 0.97 [0.78–0.93], *p* = 0.004) were associated with FA while chronotype (OR 0.89 [0.81–0.97], *p* = 0.006) and snoring (OR 1.12 [1.03–1.22], *p* = 0.01) were associated with MD (Table 3 and eTable 8, links.lww.com/WNL/D422). After further adjusting for vascular risk factors, napping showed strong evidence of a weak association with WMH (OR 1.05 [1.03–1.07], *p* = 1.49 × 10^−6^), FA (OR 1.13 [1.04–1.23], *p* = 0.005), MD (OR 1.14 [1.05–1.24], *p* = 0.002), and PSMD (OR 1.03 [1.02–1.05], *p* = 2.84 × 10^−4^) (eTable 9). People with an evening chronotype were more likely to have a higher MD (OR 0.9 [0.83–0.98], *p* = 0.017). However, other associations were no longer statistically significant. Sensitivity analyses with further adjustment for major depression and atypical antipsychotics medication usage showed similar results (eTable 10).

**Table 3:** Association of Each Sleep Variable With Each MRI Marker of SVD Both After Controlling for Age, Sex, and Ethnicity and After Controlling for Both Demographic and Cardiovascular Risk Factors

Sleep measures	Outcome	N	Controlling for age, sex, and ethnicity			Controlling for demographic and cardiovascular risk factors		
			β	SE	*p* Value	β	SE	*p* Value
Sleep duration	WMH	41,535	0.0001	0.0045	0.982	0.002656	0.004513	0.556
Insomnia	WMH	41,498	0.0241	0.0107	0.024	0.007863	0.010788	0.466
Chronotype	WMH	37,199	−0.0177	0.0097	0.067	−0.01322	0.009789	0.177
Napping	WMH	41,511	0.0688	0.0095	5.56E-13	0.046697	0.009702	1.49E-06
Dozing	WMH	41,459	0.0293	0.320545	0.360	0.021213	0.032208	0.510
Snoring	WMH	39,200	−0.0284	0.009977	0.004	0.02211	0.010349	0.0327
Sleep duration	FA	40,643	0.0038	0.0202	0.849	0.00949	0.020239	0.639
Insomnia	FA	40,624	0.1488	0.04801	0.002	0.091216	0.048207	0.0585
Chronotype	FA	36,398	−0.0919	0.043747	0.036	−0.07045	0.044017	0.109
Napping	FA	40,636	0.2129	0.042946	7.21E-07	0.121745	0.043419	0.005
Dozing	FA	40,584	0.1631	0.144319	0.258	0.109374	0.144243	0.448
Snoring	FA	38,379	−0.1612	0.045017	0.0003	0.011804	0.046441	0.799
Sleep duration	MD	40,643	0.0393	0.02	0.050	0.03174	0.02013	0.115
Insomnia	MD	40,624	0.0429	0.047696	0.368	0.02187	0.04796	0.648
Chronotype	MD	36,398	−0.1198	0.043388	0.006	−0.10413	0.043717	0.017
Napping	MD	40,636	0.1550	0.04267	0.0003	0.132881	0.043196	0.002
Dozing	MD	40,584	0.0858	0.143382	0.550	0.101172	0.143508	0.826
Snoring	MD	38,379	0.1146	0.044676	0.010	0.148667	0.04615	0.001
Sleep duration	PSMD	40,364	0.0025	0.004292	0.559	0.005335	0.004328	0.218
Insomnia	PSMD	40,345	0.0003	0.010219	0.979	−0.01268	0.010309	0.219
Chronotype	PSMD	36,155	−0.0058	0.009292	0.530	−0.00256	0.009393	0.785
Napping	PSMD	40,357	0.0511	0.009144	2.26E-08	0.033715	0.009289	0.0003
Dozing	PSMD	40,306	0.0236	0.030695	0.442	0.01445	0.030825	0.639
Snoring	PSMD	38,122	−0.0116	0.009575	0.226	0.024362	0.009924	0.014

Abbreviations: FA = fractional anisotropy; MD = mean diffusivity; PSMD = peak width of skeletonized mean diffusivity; SVD = small vessel disease; WMH = white matter hyperintensity.

### MR Analyses

**Figure 2:**
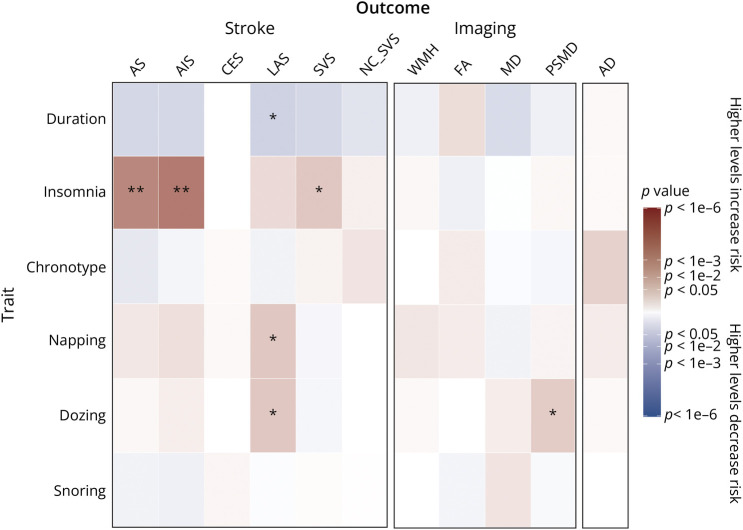
MR Results for the Association Between Sleep Measures With Clinical End Points (Stroke, Dementia) and SVD Imaging Traits Red indicates a positive β coefficient while blue indicates a negative β coefficient. * indicates that the results were significant before FDR correction, and ** indicates that the results remained significant after FDR correction for multiple comparisons. AD = Alzheimer disease; AIS = all ischemic stroke; AS = all stroke; CES = cerebral embolic stroke; FA = fractional anisotropy; FDR = false discovery rate; LAS = large-artery stroke; MD = mean diffusivity; NC_SVS = neuroimaging-confirmed lacunar stroke; PSMD = peak width of skeletonized mean diffusivity; SVD = small vessel disease; SVS = small vessel stroke; WMH = white matter hyperintensity.

MR analyses found no significant association of genetically determined napping and dozing with AS, ischemic stroke, SVS, neuroimaging-confirmed lacunar stroke, cardioembolic stroke, and AD (all *p* > 0.2, Figure 2, eTable 11, links.lww.com/WNL/D422). Genetically elevated propensities for napping and dozing were associated with higher risk of large-artery stroke (OR 1.90 [1.04–3.47], *p* = 0.035 for napping; OR 3.47 [1.09–16.57], *p* = 0.037 for dozing), but the results were no longer significant after FDR correction. Genetically elevated levels of insomnia were associated with increased risk of AS (OR 1.27 [1.10–1.47], *p* = 0.00072) and AIS (OR 1.31 [1.13–1.51], *p* = 0.0003) after FDR correction. Insomnia was also significantly associated with SVS (OR 1.56 [1.03–2.36], *p* = 0.03), but not after FDR correction. No reverse causality was observed for either AS (*p* = 0.56) or AIS (*p* = 0.19) (eTable 12).

There were no statistically significant associations between any sleep measures and WMH, FA, or MD. All other results were not statistically significant. There was no evidence for pleiotropy (eTable 11, links.lww.com/WNL/D422), and the MR-PRESSO distortion test detected 1 outlier for insomnia and AS and 2 outliers between insomnia and ischemic stroke. The outlier-corrected results were still statistically significant (OR 1.28 [1.17–1.49], *p* = 0.0006 for AS; OR 1.32 [1.14–1.53], *p* = 0.0002 for AIS). All MR-PRESSO global test, outlier test, distortion test, and outlier-corrected results are summarized in eTables 13–16.

## Discussion

We investigated the relationship of sleep with stroke and dementia, using both observational and genetic data, in over 500,000 individuals. Our observational study found associations between multiple sleep measures and both stroke and dementia, as well as associations between napping and snoring with SVD imaging traits. The association of insomnia with stroke was confirmed in our MR analyses.

In longitudinal analyses evaluating whether sleep measures led to incident stroke and dementia, the results revealed that insomnia, chronotype, daytime napping, daytime sleepiness, and snoring were associated with AS and ischemic stroke, suggesting a strong relationship between sleep and stroke, consistent with previous evidence.^26-28^ After adjusting for confounders and vascular risk factors, the associations for insomnia, daytime napping, and dozing attenuated but remained statistically significant, in accordance with previous work which found that daytime napping was associated with increased risk of stroke.^29^ We also found that sleep duration, chronotype, daytime napping, daytime sleepiness, and snoring were associated with all-cause dementia, which is consistent with many previous studies associating sleep duration, chronotype, daytime napping, and daytime sleepiness with dementia risk.^30-33^ However, after adjusting for vascular risk factors, only the associations with daytime sleepiness remained statistically significant. This suggests that the relationship between sleep measures and dementia risk may be mediated by conventional cardiovascular risk factors such as blood pressure, smoking, and alcohol consumption. Although we found a statistically significant association of increased insomnia with reduced risk of AD in the longitudinal analyses, there was no evidence to support a causal association because the MR analyses showed only a weak association of genetically determined insomnia with increased risk of AD which was not statistically significant. Although the analyses were adjusted for a wide range of potential confounders and vascular risk factors, there could still be other confounders that may have led to the observed association in the longitudinal analyses.

In view of recent hypotheses that sleep is a risk factor of SVD and that this could partially mediate the associations between sleep and dementia, possibly through the glymphatic system, we examined associations between sleep and MRI markers of SVD in over 40,000 individuals with available brain MRI scans. As well as evaluating the conventional marker WMH, we also examined associations with DTI measures of white matter ultrastructure; such measures have been shown to be more strongly associated with cognitive impairment than WMH.^13,34^ FA measures directionality of diffusion, MD measures the extent of diffusion, and PSMD is an automated metric that measures MD within the white matter tracts.^13^ Fewer associations than with stroke and dementia persisted after controlling for cardiovascular risk factors, although daytime napping was consistently associated with WMH and all DTI measures after adjusting for vascular risk factors, which aligns with previous findings.^35^ Snoring was associated with both diffusivity measures, MD and PSMD.^27,28^ This suggests that napping and snoring may be risk factors of SVD, although the analysis was cross-sectional and needs to be replicated in a longitudinal study to reduce risk of confounding.

To further investigate the causal nature of these associations, our MR analyses found evidence of causal associations linking genetically determined insomnia to risk of stroke and ischemic stroke. Our MR results did not support causal relationships of genetically determined daytime napping, sleepiness, and snoring with stroke, dementia, and imaging markers, indicating that these relationships may be confounded by other variables. Our MR analysis did not support a causal association between sleep characteristics and dementia, which is consistent with a previous study.^36^ There was no evidence of a causal relationship of genetically determined insomnia with SVD imaging markers.

Several reasons might explain the lack of significant associations in the MR analyses for daytime napping and dozing. Confounding factors may have played a role in the identified associations in the observational study. Although our study included demographics and vascular risk factors, it is possible that other implicit confounders were not accounted for.^37^ In addition, the possibility of reverse causality, where stroke survivors or patients with SVD experience increased daytime sleepiness and napping, cannot be ruled out.^32^ It is possible that patients surviving from stroke have increased levels of daytime sleepiness and napping.^30^

Previous studies have implicated sleep apnea as a risk factor of stroke,^38^ and snoring may be indicative of sleep apnea syndrome. However, we found no associations of snoring with stroke in either the observational or MR analyses. The previously reported association might be confounded by other cardiovascular comorbidities such as hypertension and type 2 diabetes, and we included controlling for these in our primary analysis. Consistent with this, previous MR studies have reported no association between sleep apnea and stroke,^39^ supporting potential confounding of the previously reported epidemiologic associations.^38^

Our study has several strengths. The use of UK Biobank enabled a very large sample size of well-characterized individuals with long-term follow-up to be analyzed, of whom over 40,000 had brain MRI scans available. We also combined both observational and MR analyses to characterize the nature of the associations and assess causality, which increased the reliability of the findings. Our study is more comprehensive than previous analyses with respect to its large sample size, the use of multiple sleep variables as exposures, and the inclusion of multiple outcome variables including stroke, dementia, and SVD markers, which may provide mechanistic insights.

However, the study also has limitations. First, most of the sleep measures were derived from self-reported questionnaires. Recently, derived accelerometry data, including measures of sleep duration, have been released in the UK Biobank, which provide more precise measures of sleep not subject to recall bias. Future work should use these data and compare the results with this study. Several factors may have also reduced the ability of MR analyses to identify associations. We used the UK Biobank for the observational analyses and large GWAS data sets including the UK Biobank and the GIGASTROKE Consortium for the genetic analyses, which provided much higher statistical power. However, some of the datasets used for the genetic associations with the sleep measures and outcomes were derived at least partially from the UK Biobank. This overlap in participants may have contributed to some degree of overfitting and weak instrument bias. The GIGASTROKE Consortium included 12% of cases from the UK Biobank for AS. However, previous MR studies that excluded overlapping participants obtained similar results to their main analyses,^19^ demonstrating that due to the large sample sizes of the respective studies, the bias due to sample overlap is expected to be very small. Another limitation is that each sleep measure has specified a time frame pertaining to the last 4 weeks. Therefore, it is possible that the questionnaire may not be capturing a long-term exposure because changes in sleep quality that occurred months or years before the baseline assessment may be relevant to long-term effects on health outcomes. Moreover, because of the lack of sufficiently large datasets for genetic associations with dementia subtypes, we were only able to conduct MR analyses using AD summary statistics from IGAP; future work should conduct MR analyses on other types of dementias, including vascular dementia and FTD, when these data become available. Although sensitivity tests were performed, the MR results may still be affected by horizontal pleiotropy and reverse causality, which is a technical limitation in this method. Finally, if the relationships are U-shaped, as was suggested for sleep duration and SVD traits,^40^ these may not be detected by MR.

The findings of this study have important implications for clinical research and practice. For dementia, our observational analyses identified multiple associations of almost all sleep measures with dementia even after controlling for cardiovascular risk factors, although none persisted in the MR analyses. This may reflect limitations in the genetic instruments we had available, but raises caution as to the causality of the observational associations. This is important because correction of sleep disorders has been suggested as preventative therapy for dementia, but our findings highlight that randomized controlled trials are required before routine sleep interventions should be recommended as a proven treatment. For stroke, we found multiple observational associations, but many of these were no longer significant after controlling for cardiovascular risk factors. However, the associations with insomnia and napping persisted after adjustment, and our MR analyses found associations of genetically determined insomnia with stroke risk and of napping with risk of large-artery stroke, supporting causal relationships. This raises the possibility that treating insomnia may reduce stroke risk and recurrence, but again, this needs testing in clinical trials. Finally, our study does not provide strong evidence that sleep disturbances are a strong risk factor of SVD or that there is a major mechanism linking sleep with dementia. Although associations with napping and snoring were identified on observational studies, our MR analyses did not confirm evidence of causal relationships.

## Appendix Authors

**Table:**

Name	Location	Contribution
Chutian Guo, BSc	Stroke Research Group, Department of Clinical Neurosciences, University of Cambridge, United Kingdom	Drafting/revision of the manuscript for content, including medical writing for content; study concept or design; analysis or interpretation of data
Eric L. Harshfield, PhD	Stroke Research Group, Department of Clinical Neurosciences, University of Cambridge, United Kingdom	Drafting/revision of the manuscript for content, including medical writing for content; major role in the acquisition of data; study concept or design; analysis or interpretation of data
Hugh S. Markus, DM	Stroke Research Group, Department of Clinical Neurosciences, University of Cambridge, United Kingdom	Drafting/revision of the manuscript for content, including medical writing for content; study concept or design; analysis or interpretation of data

## Glossary

1KG: 1000 Genomes
AD: Alzheimer disease
AIS: all ischemic stroke
AS: all stroke
DTI: diffusion tensor imaging
FA: fractional anisotropy
FDR: false discovery rate
FTD: frontotemporal dementia
GWAS: genome-wide association study
HR: hazard ratio
ICH: intracerebral hemorrhage
IGAP: International Genomics of Alzheimer's Project
IQR: interquartile range
IVW-MR: inverse-variance weighted univariable MR
MD: mean diffusivity
MR: Mendelian randomization
MR-PRESSO: MR pleiotropy residual sum and outlier
NC_SVS: neuroimaging-confirmed lacunar stroke
OR: odds ratio
PSMD: peak width of skeletonized mean diffusivity
SVD: small vessel disease
SVS: small vessel stroke
WMH: white matter hyperintensity
